# Maintaining human milk bank services throughout the COVID‐19 pandemic: A global response

**DOI:** 10.1111/mcn.13131

**Published:** 2021-01-06

**Authors:** Natalie Shenker, Marta Staff, Amy Vickers, Joao Aprigio, Satish Tiwari, Sushma Nangia, Ruchika Chugh Sachdeva, Vanessa Clifford, Anna Coutsoudis, Penny Reimers, Kiersten Israel‐Ballard, Kimberly Mansen, Radmila Mileusnic‐Milenovic, Aleksandra Wesolowska, Johannes B. van Goudoever, Mohammadbagher Hosseini, Daniel Klotz, Anne Hagen Grøvslien, Gillian Weaver, Virtual Collaborative Network of Milk Banks and Associations

**Affiliations:** ^1^ Department of Surgery and Cancer Imperial College London London UK; ^2^ Human Milk Foundation Rothamsted Institute Hertfordshire UK; ^3^ The Centre for Simulation, Analytics and Modelling (CSAM) University of Exeter Business School Exeter UK; ^4^ Mothers' Milk Bank of North Texas; Human Milk Bank Association of North America Fort Worth Texas USA; ^5^ Ibero‐American Human Milk Bank Program, National Milk Bank Service of Brazil, Fernandes Figueira Institute, Oswaldo Cruz Foundation – FIOCRUZ Ministry of Health – Brazil Brasília Brazil; ^6^ Human Milk Banking Association of India Dr Punjabrao Deshmukh Memorial Medical College Amravati India; ^7^ National Human Milk Bank, Department of Neonatology Lady Hardinge Medical College & Kalawati Saran Children's Hospital New Delhi India; ^8^ Vatsalya Maatri Amrit Kosh ‐ the National Comprehensive Lactation Management Centre, Department of Neonatology Lady Hardinge Medical College and Kalawati Saran Children's Hospital New Delhi India; ^9^ Maternal Newborn Child Health and Nutrition, PATH India New Delhi India; ^10^ Australian Red Cross Lifeblood Milk West Melbourne Victoria Australia; ^11^ HMBASA (Human Milk Banking Association of South Africa) South Africa; ^12^ School of Clinical Medicine, University of KwaZulu‐Natal Durban South Africa; ^13^ HMBASA, iThembu Lethu Community Milk Bank Rossburgh South Africa; ^14^ Maternal, Newborn, Child Health and Nutrition PATH Seattle Washington USA; ^15^ First Serbian Human Milk Bank Institute of Neonatology Belgrade Serbia; ^16^ Laboratory of Human Milk and Lactation Research, Regional Human Milk Bank in Holy Family Hospital, Department of Medical Biology Medical University of Warsaw Warsaw Poland; ^17^ Dutch National Human Milk Bank, Amsterdam UMC University of Amsterdam, Emma Children's Hospital Amsterdam The Netherlands; ^18^ Department of Neonatology Tabriz University of Medical Sciences, Neonatal and Perinatal Department, Alzahra Teaching Hospital Tabriz Iran; ^19^ Full Professor of Neonatology, Pediatric Health Research Center, Tabriz University of Medical Sciences Tabriz Iran; ^20^ Center for Pediatrics, Division of Neonatology and Pediatric Intensive Care Medicine, Medical Center – University of Freiburg, Faculty of Medicine University of Freiburg Freiburg Germany; ^21^ Milk Bank Manager, Norwegian Accredited Breastfeeding Consultant, Multi‐cultural Healthcare Consultant, Department of Pediatrics Oslo University Hospital Oslo Norway

**Keywords:** breastfeeding, COVID‐19, donor human milk, infant feeding, milk bank, nutrition, pandemic, prematurity

## Abstract

If maternal milk is unavailable, the World Health Organization recommends that the first alternative should be pasteurised donor human milk (DHM). Human milk banks (HMBs) screen and recruit milk donors, and DHM principally feeds very low birth weight babies, reducing the risk of complications and supporting maternal breastfeeding where used alongside optimal lactation support. The COVID‐19 pandemic has presented a range of challenges to HMBs worldwide. This study aimed to understand the impacts of the pandemic on HMB services and develop initial guidance regarding risk limitation. A Virtual Collaborative Network (VCN) comprising over 80 HMB leaders from 36 countries was formed in March 2020 and included academics and nongovernmental organisations. Individual milk banks, national networks and regional associations submitted data regarding the number of HMBs, volume of DHM produced and number of recipients in each global region. Estimates were calculated in the context of missing or incomplete data. Through open‐ended questioning, the experiences of milk banks from each country in the first 2 months of the pandemic were collected and major themes identified. According to data collected from 446 individual HMBs, more than 800,000 infants receive DHM worldwide each year. Seven pandemic‐related specific vulnerabilities to service provision were identified, including sufficient donors, prescreening disruption, DHM availability, logistics, communication, safe handling and contingency planning, which were highly context‐dependent. The VCN now plans a formal consensus approach to the optimal response of HMBs to new pathogens using crowdsourced data, enabling the benchmarking of future strategies to support DHM access and neonatal health in future emergencies.

**Key messages**

- Milk banking services are highly vulnerable to new infectious pathogens.
- Early in the COVID‐19 pandemic, a Virtual Communication Network was established to collect data and experiences from milk banks across 35 countries.
- Data collected estimates over 800,000 infants worldwide receive donor human milk yearly, with ~500,000 infants born <32 weeks lacking access
- Seven pandemic‐related vulnerabilities in service provision were identified, including maintaining sufficient donors, transport logistics, safe handling, and contingency planning. Mitigations are proposed.
- The VCN now seeks to build upon this work to inform and improve future responses as the Global Alliance of Milk Banks and Associations.

## 1 INTRODUCTION

If mother's own milk (MOM) is not available for low birthweight or otherwise vulnerable infants, donor human milk (DHM) from a human milk bank (HMB) is recommended by the World Health Organization (WHO), United Nations Children's Fund (UNICEF) (World Health Organization, 2011; World Health Organization/United Nations Children's Fund, 1980) and many national bodies (AAP Section on Breastfeeding, 2012; Arslanoglu et al., 2013; Mizuno et al., 2020) as the next best option for achieving exclusive human milk diets and ensuring optimal nutrition.

Throughout the COVID‐19 pandemic, the use of DHM where breastfeeding was not possible has been promoted by the WHO (2020a). Recent published data on viral infectivity from samples of women with confirmed COVID‐19 have confirmed that there is no evidence that SARS‐CoV‐2 can be transmitted via breastmilk (Chambers, Krogstad, Bertrand, Conteras, et al., 2020), supporting epidemiological evidence that there is minimal evidence of breastfeeding being a route of vertical transmission (Renfrew et al., 2020). However, milk banks are facing considerable challenges during the pandemic in maintaining the operation of services, alongside uncertainty in terms of which additional practices, if any, should be introduced into milk bank processes to maintain safety. Many of these challenges are related to external forces, such as from the impact of national pandemic responses impacting donor recruitment, staffing numbers and logistics, lack of internationally agreed minimum standards, and increased demand related to the pandemic, rather than safety challenges.

In this assessment, we aimed to estimate the scale of HMB services globally, outline the challenges facing provision of donor human milk in the context of a global pandemic, describe how HMBs worldwide are working rapidly together to mitigate them and highlight service vulnerabilities that require greater investment to ensure that exclusive human milk diets for vulnerable neonates can be maintained in this and future emergencies.

## 2 METHODS

### 2.1 Creation of the Virtual Collaborative Network

The core Virtual Collaborative Network (VCN) was formed over a 2‐month period from 17 March, just as the WHO declared a global pandemic. It was formed by using a WhatsApp group, which the founders G. W. and N. S. recognised was a technology available in every country, without censorship and available to anyone with a mobile phone. As such, the founders approached the heads of every milk bank association that represented milk banks in more than one country, as well as milk bank leads from countries where individual milk banks operated (e.g. Kenya). We also approached nongovernmental organisations (PATH and Alive and Thrive) who had expertise in this sector with links to the WHO, in order to facilitate the recruitment of milk bank leads into the VCN and information flow between the VCN and WHO. Academics with specific expertise in human milk banks, including neonatologists who were clinical directors for milk banks, and social scientists, including anthropologists, were approached to join by email, followed up with a link to the WhatsApp group. In the first 2 months, a weekly update was made available on a central Google Doc resource so that new members to the VCN could review the conversations and information that had already been exchanged by the group. In a similar manner, this manuscript was effectively ‘crowdsourced’ as all members had access to edit and submit country‐ or regional‐specific information.

### 2.2 Estimation of the scale of human milk banking globally

Predictions were made of the total number of premature recipients across the countries with operational HMBs. This was done by making use of publicly available per‐county birth rates (Central Intelligence Agency, 2020), UN estimates of population sizes (Worldometer, 2020) and preterm birth rates per region (Blencowe et al., 2012). Mortality rates were not factored in. Results were generated on a regional basis, with designations of countries to regions as specified by Blencowe et al. For the purposes of this estimation, the population of preterms considered include births below 32 weeks gestational age. An initial number of HMBs per country was obtained from PATH (2020), updated where necessary from information provided by members of the VCN local to those countries.

VCN members were requested to share up to date information regarding HMB operations (numbers of recipients and DHM volumes). The granularity of the provided data varied. Regional data were obtained for instance in the case of North America (via Human Milk Banking Association of North America), and national data were received for several countries, for example, Brazil and India; otherwise, data were received for individual HMBs. As the information received was not complete, that data were only made available for a subset of countries/or individual HMBs within a country, and the number of recipients was not provided in many cases; approaches for estimating the missing data were required. Three such approaches were employed: (A) for countries where only the volume of DHM is reported, the number of recipients is estimated using the volume per recipient, averaged over all (global) responses that included the volume and number of recipients; (B) for countries reporting data for only a subset of known HMBs, data were extrapolated to the full set of known HMBs within that country; (C) for countries for which only the number of HMBs was known, the number of recipients was estimated based on the (global) average of the calculated number of recipients per HMB where data allows. Data from each milk bank leader and national associations (>80 members of the VCN as of 1 May) were collated by three authors (M. S., N. S. and G. W.). Data were collated and analysed using Excel (Microsoft 365, Microsoft, WA).

### 2.3 Qualitative data collection

A set of open‐ended questions were circulated to the group to ask for their experience of operating an HMB, or the experiences of a national or regional network, in approximately the first 2 months of the global pandemic. Evidence collection started on 23 March and concluded on 1 May. Experiential descriptions of challenges faced in milk bank service provision during the COVID‐19 pandemic were submitted to and analysed by G. W. and N. S. for themes regarding the challenges raised by the COVID‐19 pandemic. Examples of responses to the pandemic were communicated by each of the co‐authors in their country‐specific context, and each co‐author read and approved the mitigation steps as outlined.

## 3 RESULTS

### 3.1 Scale of human milk bank services

As of 22 June 2020, information was obtained from 32 countries out of the 66 countries with known operational HMBs (PATH, 2020), indicating that there are currently 756 operational HMBs. Actual data regarding recipient populations were made available from 42.1% of HMBs, with further estimates available from 16.9% (Table 1). The recipient population, average duration and volume of DHM provision varied by setting according to figures provided by or estimated for each region (Table 1). The estimated average volume of DHM per recipient worldwide was 710 ml. However, DHM provision is nonuniform, even within single countries, and the range was extremely wide. For example, the estimated average volume of DHM per recipient in India according to the National Milk Bank Service of India was ~230 ml, but detailed data from a single HMB in India estimated that the average volume of DHM per recipient is <100 ml per infant, with this volume serving as a bridge to full maternal milk provision. This contrasts with availability and provision of donor milk found in countries within high‐income countries. In Norway, where breastfeeding initiation rates are high (Victora et al., 2016), the average volume per recipient was over 3 L of DHM, reflecting the much wider criteria for use (i.e. term and sick infants in hospital), as well as greater DHM availability. The figures provided for the represented countries in the Latin America region show that current provisions for DHM exceeds that required for infants born below 32 weeks GA (Table 1), suggesting that older infants are also receiving DHM, which would not be true for other regions.

**TABLE 1:** Number of projected current recipients in hospital settings (unspecified GA) and number of predicted premature births of <32 weeks GA, per region per year for countries with HMBs

Region^a^	No. of operational HMBs	HMBs providing data provided	Actual or estimated recipients provided (no. of HMBs)	Estimated recipients (*n*, HMBs)^b^	Average recipients yearly per HMB	Preterm births <32 weeks GA^c^
Northern Africa and Western Asia	Not known	n/a	n/a	None known	n/a	21,698
Latin America and the Caribbean	306	224	224	261,334	854	139,623
Developed	289	153	76	381,008	1318	171,070
Central and Eastern Asia	28	16	15	5433	194	211,566
South‐Eastern Asia and Oceania	25	4	3	4659	186	123,946
Sub‐Saharan Africa	24	1	0	35,944	1498	88,528
Southern Asia	84	48	0	117,147	1395	575,208
Total	756	446	318	805,524		1,331,639

^a^ As defined in Blencowe et al. (2012).

^b^ Three approaches were employed for estimation where data were missing. (A) For countries where only the volume of DHM is reported, then the number of recipients is estimated using the volume per recipient, averaged over those responses (which included the volume and number of recipients). (B) For countries reporting data for only a subset of known HMBs, data were extrapolated to the full set of known HMBs within that country. (C) For countries for which only the number of HMBs was known, the number of recipients was estimated based on the average of the calculated number of recipients per HMB where data allows.

^c^ Based on statistics of population and birth rates per country with operational and planned milk banks, and proportion of preterm births per region for GA < 32 weeks (Blencowe et al., 2012).

From the 446 HMBs for which data were made available, projections were made for the remaining 310 out of all known 756 HMBs. The results indicated that approximately 806,000 babies in hospital receive DHM annually worldwide (Table 1). If this estimate is correct, it is possible that >50% of babies born before 32 weeks GA would have access to DHM in countries with HMBs (806,000 out of 1.3 million babies born before 32 weeks) (Blencowe et al., 2012). This finding is also likely to be an optimistic estimate as in some settings, access to donor milk, feeding policies and lactation support overall is suboptimal. Nevertheless, these figures still indicate that a significant overall shortfall in availability of DHM worldwide is likely.

### 3.2 COVID‐19 related challenges to HMB service provision and safe operational processes

The experiences from milk banks across 35 countries were collected and revealed the following challenges. The major challenges spanned seven themes, briefly summarised below and developed HMB practice recommendations (below and Table 2). Representative country‐specific reports are provided in Data S1.

**TABLE 2:** Core themes and preliminary mitigations for the safeguarding of donor human milk provision (adapted from Shenker, Hughes, Barnett, & Weaver, 2020)

Challenge	Mitigation
Supporting mothers to provide MOM	Milk banks should operate in the context of optimal lactation support, ensuring that minimal quantities of DHM are used without undermining maternal breastfeeding. The VCN is developing a programme of education to ensure that this ethos underpins the work of milk banks globally.
Donor engagement	Traditional media (radio, print and TV) and social media calls have been successfully employed to ensure supply of donor milk continues.
Additional screening of milk donors	Each milk bank now uses a set of prescreening questions on symptoms, test results and exposure to COVID‐19. Donors should delay donation or expressing and storing milk for donation until asymptomatic or may be deferred permanently. The exclusion also applies if the donor is a known contact of someone with these symptoms, or a COVID‐19 diagnosis 14 days after contact.
Serological screening	As a result of COVID‐19 restrictions, potential milk donors are finding it increasingly difficult to access phlebotomy services for their compulsory screening tests. Milk banks have responded in various ways, including working with volunteer phlebotomists (e.g. Scotland), travelling to donor homes within a 50 mile radius (e.g. Chester) and working with the NHSBT to access phlebotomy at Donor Centres (e.g. Hearts). Donor recruitment has also focussed on mothers of inpatient infants, who could get bloods taken in the hospital.
Communication	Milk banks have not only engaged in greater levels of communication with each other but also to donors and community‐based recipient families, to reduce uncertainty and stress. For NICUs, regular communication is also essential: (i) to determine levels of demand and changes to infant‐feeding policies affecting DHM use; and (ii) for units to be informed about any potential interruption of DHM supply.
Collection and transportation of donor milk	Noncontact collection‐and‐delivery processes have been implemented and HMBs are working rapidly to adapt to these new measures while adhering to all aspects of screening and quality control. Donors should be screened before face‐to‐face contact according to the additional screening questions suggested above. Social distancing, no‐contact collections and deliveries to units and appropriate personal protection equipment (PPE) use should be observed where appropriate by donors, staff, volunteers and couriers engaged on behalf of HMBs. A suggested Standard Operating Procedure was developed by the Hearts team which is now adopted nationally.
Donor milk handling	SARS‐CoV‐2 can maintain infectivity while on plasticware, stainless steel, and cardboard for several hours/days, (Chin et al., 2020; Kampf, Todt, Pfaender, & Steinmann, 2020) but this is not a major transmission route. There is therefore a small risk of accidental transmission during container handling if standard protocols for DHM handling are breached. Typically, hygiene and handwashing are highly stringent in HMBs. There is no evidence to support the use of disinfection of containers, and this approach may introduce a secondary risk of feed contamination with bleach or other viricides.
Milk quarantine	Some HMBs are instituting milk quarantine, whereby prepasteurised milk is kept separately from other stocks for 14 days after the last expression. Before milk is removed from the freezer, donors are contacted to ensure they have been symptom‐free for the previous 14 days. However, this is guidance only, and in no way should compromise stocks of donor milk if sufficient supplies are not in stock.
Contingency planning	HMBs are generally under‐resourced and minimally staffed, operate without a large DHM surplus, and risk closure due to self‐isolation. Each HMB is now actively considering contingency plans for which HMBs could cooperate to safeguard supplies.
Safety of milk bank staff	Additional measures should be taken by milk bank staff who are in contact with donors by wearing situation appropriate PPE, particularly given asymptomatic transmission is likely.

#### 3.2.1 DHM availability

In some settings, breastfeeding policies have led to an increased demand for DHM. The Chinese Expert Consensus Group reported that infants who were symptomatic or positive for COVID‐19 should be cared for in a quarantine room and fed DHM for 14 days (Wang et al., 2020). Such recommendations influenced hospital neonatal unit decision‐making beyond China and placed additional pressures on HMB services. Even the largest national HMB network in Brazil (established in 1998 with 224 active HMBs and 215 milk collection centres) encountered low supplies of DHM as a result of decreased donations and increased demand. Pressure on DHM supplies related to notable differences in how mothers donate surplus breastmilk to HMBs, including those who (i) have low birthweight or sick infants in hospital; (ii) are at home and donating a one‐off store of milk expressed while their infant is in the NICU; (iii) are at home and are recruited to prospectively collect milk for donation; and (iv) are bereaved. From reports from the VCN, donated milk volumes can vary from 50 ml as a single donation (India) to >100 L of milk (US, UK and others) over several weeks or months. Different scenarios elicit different challenges during COVID‐19 restrictions, each impacting a donor's ability to donate her surplus milk to an HMB.

To avoid overcrowding during the COVID‐19 pandemic, we closed the LMC as well as the Baby Clinic from 1st April 2020, which usually opened weekly. We still collect the milk from the postnatal wards. (Myanmar)

Some HMBs scaled‐up their activities and recruited more donors. The largest milk bank service in the world, the National Milk Bank Service of Brazil, urgently launched a media appeal across social, print, television and radio to encourage milk donors to donate and ensure donors were aware of the safest ways to express and store their surplus milk (J Aprigio, personal communication, May 16 2020). Elsewhere there was an overwhelmingly positive response from mothers offering their milk, as noted in the USA, UK, India, France and the Netherlands.

There has been no change to DHM services in Norway so far and most of the DHM continues to be fed raw (i.e. without pasteurisation). We have sufficient DHM for NICUs and a sufficient number of donors. More potential donors are contacting us, wanting to help. (Norway)

However, some countries (e.g. China, Poland and India) noted a reduction in the number of potential milk donors, possibly resulting from fear about leaving the house to be screened or to donate milk, lockdowns, early hospital discharges and discontinuation of outpatient services.

Generally in the first 2–3 days, milk production is less in these mothers (on the NICU), so we provide DHM to bridge the gap and then once the mothers start lactating sufficiently, babies go back to being fed mothers' milk. Right now, DHM supplies are deficient. We outsource the milk culture, and so as a result of the lockdown and lack of transportation getting milk to the lab is an issue. We have had to completely stop collecting milk. But with PATH's support we are as of yesterday collecting and storing milk again (can be stored up to three months) and we will begin the culture process once things get back to normal. We are rationing the DHM for very small and sick babies. The number of deliveries have also reduced by 35–40%, so demand has lessened too. Mothers are scared and want to avoid hospital visits as much as possible. Health care workers are facing challenges – there are knowledge gaps and lack of awareness on guidance on infant feeding. PPE deficiency is also a problem though not as widely. Our key message is that breastfeeding should not be stopped because of Covid challenges and every effort should be made to ensure the needy babies receive DHM. so proper rationing should be practiced. (India)

In some areas, demand for donor milk from hospital neonatal units decreased, likely as a result of a lack of evidence dissemination about SARS‐CoV‐2 inactivation during DHM pasteurisation and fears of transmission. In the USA, India and Iran, some hospital neonatal units applied stricter rationing of DHM (Furlow, 2020); for example, in Iran, only babies with birthweights less than 1.2 kg are now receiving DHM rather than the usual policy of infants <1.5 kg.

There is DHM in stock; however, due to logistical and hygienic reasons, its use has been limited during the pandemic. Medical staff in hospitals wish to avoid unnecessary risks, especially with the current shortages of PPE. Supporting lactation in COVID‐19 infected mothers during the pandemic is considered an unnecessary risk. (Poland)

… the need and use decreased much. It is not difficult for us to find donors because the donors who come to our human milk bank have large amounts of milk. Donor screening became very strict, like epidemiological history inquiry, commitment signature, temperature measurement, and previous examination items. (China)

#### 3.2.2 Prescreening for COVID‐19 exposure in milk donors

Although there is now a consensus that SARS‐CoV‐2 is not transmissible via breast milk, all milk banks were highly concerned that strong precautionary processes were implemented as quickly as possible to protect vulnerable infants receiving DHM from any risk of infection as well as to protect staff and volunteers. This includes identifying donors infected with (or at risk of) COVID‐19.

There is heightened screening of all mothers at the entrance to the hospital and during the process of admission. The screening also includes all the visitors to the hospital and the people accompanying mothers to the hospital. In addition to mothers being screened prior to admission, donor mothers are also getting additional COVID‐19 screening questions at the point of recruitment. (Kenya)

Additional questions were rapidly incorporated into screening and national guidelines in every country to attempt to identify donors exhibiting symptoms of COVID‐19 or who had tested positive for SARS‐CoV‐2; screening procedures were often implemented in parallel with local blood‐transfusion services.

#### 3.2.3 Logistics of milk donor serological screening

Usually, potential milk donors are required to undergo routine serological screening testing for blood‐borne viruses and other infectious pathogens. These vary according to the type of test, infections screened for, and the timing of the tests. In the UK, *Clinical Guideline #93 Donor Milk Banks: service operation*, which is used widely in Europe and beyond, from the National Institute for Health and Care Excellence recommends serological screening at the time of recruitment (National Clinical Guideline Centre [NICE], 2010). As a result of COVID‐19 restrictions, potential milk donors are finding it increasingly difficult to access phlebotomy services because they are closed or have been redirected, or potential donors were fearful of accessing these facilities.

We have local donors dropping off human milk at our facility, but most donation is by overnight shipment. We have suffered cancellations of milk drives in various communities in California. However, our hospital‐based milk collection sites/depots continue to collect milk or provide shipping containers to mothers. The biggest fear is going outside the home and becoming infected during phlebotomy. (USA)

HMBs encountering difficulties recruiting donors are learning actively from countries with different screening systems to navigate local ‘roadblocks’ while maintaining safety standards. Screening advisors discussed using antenatal screening tests rather than the current mandatory postnatal tests upon recruitment, but the VCN considered this to be below the minimum standard, considered only if stocks of DHM became critically low and only after approval by local governance structures. Milk banks also focussed on recruiting donors whose babies were in the hospital neonatal unit, where blood tests could be performed onsite by hospital neonatal unit teams. Screening difficulties can lead to novel opportunities and partnerships.

A call for donors and volunteer phlebotomists to support our milk bank was put out through social media and the BBC, with an excellent response, but sufficient numbers of donors have come through without needing to adopt unusual measures at the moment. The National Health Service Blood Transfusion Service in the UK, which has never previously worked formally with HMB services, allowed potential milk donors undergoing screening for donation to the Hearts Milk Bank to access phlebotomy services. This strategy may open future opportunities for the two services to operate more closely beyond the pandemic. (United Kingdom)

#### 3.2.4 Communication by HMBs

As new evidence emerged around safe handling and processing of human milk in the context of COVID‐19, communication was vital to reassure parents, the community and healthcare providers that DHM remains safe. Some donors reported increased levels of concern that they may transmit SARS‐CoV‐2 via their donated milk. Social media was a useful tool to disseminate updates and guidance for milk donors. Additionally, systems to improve communications and networking between HMBs are needed to ensure rapid mechanisms to share learnings, protocols and experiences, related to COVID‐19 safety. As the COVID‐19 pandemic developed, individual countries published their own guidance for the care of mothers and their babies in the perinatal period. Also, in the absence of global safety standards, HMB associations and individual HMB programmes published their own specific guidance relating to DHM in the context of COVID‐19 for regional areas (European Milk Bank Association, 2020; Human Milk Banking Association of North America [HMBANA], 2020b). The VCN itself was integral in sharing information rapidly to milk bank leaders globally.

Most information is currently anecdotal or from personal communication and needs to be systematically collected to assess the extent of potential unintentional collateral damages of the COVID‐19 response in this regard. (Germany)

#### 3.2.5 DHM collection and transportation

The COVID‐19 pandemic has led some governments to impose social distancing measures that impacted on the collection and delivery of DHM, preventing donor mothers from reaching ‘milk drop’ sites or couriers from reaching residences. Furthermore, basic transport infrastructure was closed in some areas, such as the ferry network in British Columbia, or air freight services where milk banks serve a large geographical area.

Noncontact collection‐and‐delivery processes were implemented by some HMBs. Some HMBs instituted ‘milk quarantine’ principles whereby prepasteurised milk is kept separate from other stocks until 14 days after the date of last expression. Before milk was removed from freezers, donors were contacted to ensure they had been symptom‐free for the previous 14–28 days.

At our HMB, in addition to meeting all the usual criteria, we are being extraordinarily cautious and ‘quarantining’ the breast milk of approved donors 3 weeks after expression. At 3 weeks, we check that the donor and her immediate family are well before dispensing pasteurised milk. A COVID‐19‐positive donor is resigned immediately and her milk discarded. We acknowledge this is an extremely cautious approach. (New Zealand)

However, the group felt that this should be a guidance only and in no way should compromise DHM stocks if supplies were compromised. This approach was also problematic in countries that screen donors in hospital before discharge, such as India, where follow‐up is limited. On discussion, the risks for formula feeding were assessed as significantly higher than the risk for babies receiving pasteurised DHM from mothers who have been screened via questioning regarding COVID‐19 symptoms and exposure. The experience of milk bank leaders from countries such as South Africa where the impacts of the initial response to HIV were most severe were a powerful reminder on the VCN that any interruption of supply would have serious implications.

#### 3.2.6 DHM handling

Respiratory pathogens are principally transmissible by aerosol or droplet dispersal but also from fomites (objects or materials likely to carry infection) and surfaces (Asadi et al., 2020). Early indications suggested that SARS‐CoV‐2 could maintain infectivity while on plasticware, stainless steel and cardboard for several hours or even days under experimental settings (Chin et al., 2020; Kampf et al., 2020), and although these have not been proven to be a major route of transmission, the threat to milk bank processes remains present (Dhillon, Breuer, & Hirst, 2020). Milk bank leaders were therefore clearly concerned about the risk of transmission from handling of containers/bags touched by an asymptomatic donor or courier in cases where standard protocols for DHM handling were breached.

Typically, hygiene and handwashing are highly stringent in HMBs. Although it has been suggested that containers and human milk storage bags during the COVID‐19 pandemic should be decontaminated with bleach or other viricides, the VCN considered that there was no evidence base to support this suggestion (Marinelli & Lawrence, 2020), and rather, it may introduce a secondary risk of chemical contamination of DHM (HMBANA, 2020a).

The group decided that HMB staff should: (i) practice regular handwashing; (ii) wear gloves whenever handling containers/bags containing donated milk; (iii) avoid touching their faces/spectacles; (iv) protect their skin from repeated exposure to soap, alcohol gel and water; (v) be encouraged to re‐moisturise their hands at the end of work (Yan et al., 2020); (vi) allow only limited access to their premises (including laboratory spaces and offices); (vii) practice SD between staff members; (viii) self‐isolate if in contact with a symptomatic individuals for 14 days; and (ix) solitary working procedures should also be considered if feasible.

Before each collection, we call the donor and report if she has presented symptoms. We keep 2 m from the donor and disinfect the ice box holding the DHM. (France)

#### 3.2.7 Contingency planning

HMBs are generally under‐resourced and staffed minimally and may operate without a DHM surplus. Surveys in UK and India suggest that most HMBs there operate with on to two staff and carry only 2–3 weeks' supply of DHM, making service continuation difficult in some settings. For some milk banks, requirements for social distancing and limitations on transport could last for weeks or months and will lead to potential shortcomings in DHM supply to hospitals. Specific issues may affect HMBs located within hospitals (e.g. infection control) that may be different from those located in external institutions (e.g. sample transportation for microbiological screening and transporting DHM to hospitals over a wide geographical area). There is currently no way to document the impact of HMBs closing, and there is a need for a multicountry assessment of the impact of HMB loss or being able to overcome logistical challenges.

Huge potential upcoming challenge for HMBs: turning down requests for DHM next week that would have been agreed last week. (USA)

Each HMB actively considered contingency plans including whether two or more HMBs within a reasonable geographical range could cooperate to safeguard supply of DHM for the most vulnerable infants. Innovation is needed to rectify the lack of a global communication platforms linking HMBs worldwide, which has limited the rapid sharing of information, data or protocols for a pandemic or other disaster response. As one beneficial effect of the VCN, milk bank services have already started to streamline their data collection and communication networks, and work is now starting that aims to simplify data collection strategies within and between countries. The importance of HMBs must be highlighted by neonatologists and other healthcare professionals to avoid a collapse in services as a consequence of access to DHM being regarded as ‘nonessential’.

## 4 DISCUSSION

The COVID‐19 pandemic has brought additional considerations and challenges for the mother–infant dyad, newborn nutrition and HMB operations. There is now strong evidence that the risk of SARS‐CoV‐2 vertical transmission through human milk is minimal (Chambers, Krogstad, Bertrand, Contreras, et al., 2020; WHO, 2020b) and milk bank procedures are effective at mitigating the theoretical risk of transmission (Chambers, Krogstad, Bertrand, Contreras, et al., 2020; Conzelmann et al., 2020; Unger et al., 2020; Walker et al., 2020). However, at the outset and in the absence of global standards or pan‐national milk bank communication networks, individual milk banks and associations had to develop practices to ensure safety and continuity of their services. We present the first activity of a new global alliance of milk banks, which aims to build communication networks, emergency preparedness and global standards in the context of rapid threat development.

The provision of DHM exists to support and promote breastfeeding. With appropriate use in the context of optimal support for lactation, a short period of DHM provision can support mothers to establish their milk supply without the need for supplementation with infant formula milk (Kair & Flaherman, 2017; Kantorowska et al., 2016; Merjaneh et al., 2020; Wilson et al., 2018). For the infant, separation from their mother limits access to maternal milk and should be avoided (WHO, 2020a). When MOM is not available, reduced availability of DHM will increase infant morbidity and mortality related to prematurity and other health conditions (El‐Khuffash, Jain, Lewandowski, & Levy, 2020; Hoban, Khatri, & Unger, 2020; Tully, Lockhart‐Borman, & Updegrove, 2004), as well as impacting parental mental health. During the COVID‐19 pandemic, HMBs are facing challenges in maintaining adequate staffing, donor recruitment, policy adjustments for the safe handling/transportation of DHM, and in some contexts increased demand when mothers and infants are separated (Furlow, 2020). It is essential that systems to ensure human milk feeding, including breastfeeding and the provision of DHM to vulnerable infants, not be inadvertently impacted by efforts to contain COVID‐19 (Brown & Shenker, 2020; WHO, 2017).

HMB capacity worldwide has increased in recent years as clinical evidence has mounted regarding the implications of early exposure to infant formula, particularly for very low birthweight babies (Boyd, Quigley, & Brocklehurst, 2007; Quigley, Embleton, & McGuire, 2019; Quigley & McGuire, 2014; Trang et al., 2018). Currently, HMBs operate in 66 countries, but accurate data regarding the true need for DHM are lacking. HMB expansion, however, has been challenged due to a lack of global guidelines on safety and operations (PATH, 2019) as well as, in some instances, a lack of regulatory framework for this unique biofluid. However, there is also the danger of over‐regulation, and the valuable experiences of countries from Brazil to South Africa, and many others demonstrate how milk banks can operate safely using simple and basic standards of operation. These experiences hold a common thread of governmental support, with ringfenced funding to support HMB services through external challenges. For example, in South Africa COVID‐19 added another burden on an already stretched service, which had already seen HMBs close as a result of equipment failures. The experience in Asia highlights that many HMB services were only able to continue through the intervention and funding of NGOs, rather than centrally funded through governments as a core part of health services. The considerable collective experience and expertise of the VCN will be instrumental in laying the groundwork for HMB regulation and optimal funding models, with the goal of making DHM available to more infants globally.

The possible need for DHM has been estimated using per‐country birth rates and preterm birth rates per region from published data (Blencowe et al., 2012), with the current report showing a significant potential shortfall. Importantly, future work must account for systems‐level challenges that simply prevent MOM reaching her infant. Data are needed to document the number of babies who lack an adequate supply of MOM and the reasons why, the number of infants who received DHM, the length of time it is required, and the volumes used (Breastfeeding Innovations Team, 2017). The worldwide incidence of neonatal deaths in 2018 was 18/1000 live births, representing an estimated 2.5 million newborns, of which ~0.85 million died due to the complications of preterm birth (United Nations Children's Fund [UNICEF], 2019). The UN Sustainable Development goals (Breastfeeding Innovations Team, 2017; UN, 2020) call for neonatal mortality to fall to 12 deaths/1000 by 2030, with particular impacts for regions that require specific resourcing (e.g. south Asia and Africa). Reduction in preterm‐associated mortality via more widespread provision of DHM, as one of the strategies to protect, promote and support breastfeeding, could be a key contributor to meeting this goal.

Worldwide, DHM is used primarily for premature babies being cared for in hospital neonatal units, as well as those who are low birthweight, critically ill, orphaned or abandoned. With regard to DHM provision, some countries have experienced an increase in demand during the pandemic (related to the increase in mothers who are ill and unable to express milk for preterm babies as well as separation of symptomatic mothers from their neonates) and others a decrease in demand (whereby fewer babies have been born preterm and clinicians in some countries were risk‐averse to using DHM). Guidelines have been published for the care of symptomatic infants and mothers in neonatal care (e.g. Italy; Davanzo et al., 2020), but most national bodies and regional policy leaders (e.g. CDC and RCPCh) have not released specific advice regarding DHM use. The WHO has advocated for re‐lactation support or DHM if breastfeeding is challenging, in line with IYCF emergency settings guidance (United Nations Children's Fund, Global Nutrition Cluster, & Global Technical Assistance Mechanism for Nutrition, 2020; WHO, 2020a). Research will be needed that ‘layers’ the relationship between attitudes towards DHM and lactation support in hospital neonatal units and the community with national cultural perceptions of breastfeeding and human milk and further supports the development of context‐specific guidelines.

### 4.1 Operational adaptation of HMBs to the COVID‐19 pandemic

HMB services operate according to guidelines set by national bodies or local organisations. Donors are screened using interviews and questionnaires based on health and lifestyle. This strategy aims to reduce the risk of microbial or other contamination of donated milk and is usually employed in addition to serological screening for common blood‐borne infections (NICE, 2010). Human milk is collected and, with a few exceptions (e.g. Norway and some German HMBs), pasteurised mostly via Holder pasteurisation (milk heated to 62.5°C for 30 min). At the outbreak of the pandemic, milk banks were guided by studies that documented complete heat inactivation of genetically similar viruses such as SARS and MERS by treatment at 60°C for 15–30 min (Darnell & Taylor, 2006; Rabenau et al., 2005; van Doremalen, Bushmaker, Karesh, & Munster, 2014). Early work showed that SARS‐CoV‐2 is inactivated by heating in a dose‐dependent manner, with viral inactivation at 10–30 min at 56°C, or 5 min at 70°C (Chin et al., 2020), and studies have now confirmed that Holder pasteurisation effectively neutralises SARS‐CoV‐2 (Chambers, Krogstad, Bertrand, Contreras, et al., 2020; Conzelmann et al., 2020; Walker et al., 2020).

Although our findings showed that there was a huge response from potential milk donors to meet the need for increased DHM provision, HMBs have an ethical duty to ensure that donors are not coerced to give milk that may be needed for their own baby; the emotional drive to donate altruistically, which many donors express openly, should not override their own safety or that of their infants (Hartmann, 2017; Israel‐Ballard et al., 2019). HMBs should, where appropriate, encourage mothers to reserve a stock of expressed milk to feed their babies in the event of extreme illness and/or separation.

Donors should be screened before face‐to‐face contact with the HMB team according to the additional screening questions suggested above. Even in countries where self‐isolation and social distancing is not imposed by the government, social distancing and facemask‐wearing should be considered and observed where appropriate by donors, staff, volunteers and couriers engaged on behalf of HMBs.

HMB staff must understand which epidemiological risk factors increase the likelihood of SARS‐CoV‐2 infection and which symptoms are suggestive of SARS‐CoV‐2 infection. Milk bank teams should then communicate this knowledge adequately to milk donors and apply this rigorously to their recruitment processes (Guan et al., 2020). For example, current guidance for screening in the UK and the Netherlands is to stop accepting milk donations for 8 days after symptoms develop, and only if fully recovered (in accordance with guidance for healthcare workers) or for 14 days after exposure to a confirmed case or symptomatic contact (Tong et al., 2020), whereas in Australia and Singapore, donors are excluded for 21 days after exposure and 28 days after full recovery, and in Thailand, symptomatic mothers are excluded for 28 days after diagnosis.

The exclusion also applied if the donor is a known contact of someone with these symptoms, or a COVID‐19 diagnosis, 14 days after contact. Donors should delay donation or expressing and storing milk for donation until asymptomatic. Following safety precautions implemented by blood transfusion services (Chang, Yan, & Wang, 2020), measures should be taken to protect milk bank staff in contact with donors by wearing situation appropriate personal protection equipment (PPE), particularly given asymptomatic transmission is likely.

Databases that enable rapid (but secure) communication with milk donors should be maintained by individual HMBs to ensure the security of personal information. HMBs should also communicate regularly with their local network of HMBs and hospital neonatal units: (i) to determine levels of demand and changes to infant‐feeding policies that could affect DHM use and (ii) to inform units about any potential interruption of DHM supply.

### 4.2 DHM and SARS‐CoV‐2—Safety considerations and role as a clinical therapy?

SARS‐CoV‐2 has been isolated from nasopharyngeal swabs, sputum, serum, semen and faeces (Li, Jin, Bao, Zhao, & Zhang, 2020; Zhang et al., 2020). However, it is highly unusual for a CoV or other respiratory virus to cross into breast milk (Schwartz & Graham, 2020). Although viral fragments have occasionally been identified (Groß et al., 2020), no cell culture studies have shown evidence of infectivity, and it is likely that virus detected in breast milk is not capable of causing infection in the infant (WHO, 2020b). Neither direct breastfeeding nor feeding of expressed human milk has been shown to be a route to vertical transmission (Renfrew et al., 2020).

The global nature of this VCN reflects advice from the WHO with regard to health systems in both developed and developing nations. HMB leaders who have lived and worked through the earliest years of the HIV pandemic bring insights into the mistakes that occurred in the 1980s, with fear of HIV discouraging mothers from breastfeeding and costing the lives of many babies who received infant formula in unsafe conditions (Moland et al., 2010). Unlike HIV where transmission of the virus via breastfeeding was a possibility, there is currently no evidence around SARS‐CoV‐2 transmission from breastfeeding or human milk. Therefore, to avoid further impacting an already strained health system during the COVID‐19 pandemic, the best chance to keep infants healthy is to normalise breastfeeding and promote its protective factors along. As DHM provision plays an important role, HMB services should be supported by governments as part of strategically aligned breastfeeding policies.

Serum antibodies against SARS‐CoV‐2 appear 5 days after symptom development, with specific IgM antibodies appearing at 10 days and IgG antibodies developing by 14 days (Chen & Li, 2020). Profiling of human milk for immunoglobulins has suggested that ~80% of mothers produce IgA, with some IgG, antibodies against SARS‐CoV‐2 (Dong et al., 2020; Fox et al., 2020). Serological screening of donor mothers may be useful as part of research into whether infants exposed to or infected with SARS‐CoV‐2 may benefit from DHM from mothers who have recovered from COVID‐19. This strategy, if shown to work, would enable antibody‐positive human milk to be targeted to hospital neonatal units to feed infants of symptomatic mothers unable to express their own milk and other vulnerable populations.

### 4.3 Virtual Communication Network

The newly established VCN has rapidly facilitated the sharing of information, discussion of evidence and development of consensus views of best practice related to local circumstances. The group is still growing and now includes over 100 members from 39 countries as of 10 July. The VCN endorses the WHO's recommendations not to separate the mother and infant and to support breastfeeding, thereby reducing the use of formula milk, while also decreasing the demand for DHM and improving outcomes for the mother and her infant. However, in the context of separation of a symptomatic mother and infant, DHM use may be a critical ‘bridge’ for the infant, assuming that systems will simultaneously provide critical lactation support to ensure the mother can initiate and maintain lactation during separation.

In this report, the VCN identified key challenges, alongside potential solutions to mitigate their impacts, which HMBs have either adopted or will consider should their local situation worsen. It can be particularly difficult for individual services, those with few staff, and those without a cohesive national framework to respond to new and urgent challenges such as the COVID‐19 pandemic rapidly and appropriately. This report not only provides recommendations but acts as a benchmark for HMB leaders worldwide to cooperate and collaborate to strengthen services in the future.

## 5 CONCLUSIONS

The COVID‐19 response to prevent infection and reduce global spread must also ensure that inadvertent harm is not done to other critical aspects of care and prevention. HMBs around the world are facing unprecedented challenges to maintain safe DHM supplies in volatile health system infrastructures that limit routine operations. Many HMB systems have struggled to respond to the COVID‐19 pandemic, with issues deepened by the lack of globally agreed safety guidelines and rapid communications for emergencies, as well as limited data and infrastructure to ensure responsiveness during a crisis. Additionally, policies designed to ensure safety between mother and infant during suspected or confirmed COVID‐19 infection have been developed rapidly and often resulted in mixed messages and confusion. The consensus from this VCN is that contact should be maintained between mothers and babies, with skin‐to‐skin and breastfeeding support. If DHM is provided, this should be for as short a time as possible as a bridge to receiving MOM, ensuring that the global supply of DHM can continue to be used for those most vulnerable, when maternal breastfeeding is not possible. Mothers are more likely to breastfeed if DHM is available and used appropriately with optimal support for lactation. Strengthening of the HMB system is required to ensure that provision of safe DHM remains an essential component of early and essential newborn care—during routine care, as well as emergency scenarios, such as natural disasters and pandemics.

COVID‐19 has presented challenges and opportunities for health systems; the HMB sector seeks to build upon the learnings from this period to inform and improve response in the future. This VCN is now focussed on building upon the key themes identified to achieve a formal consensus and set of activities that can assist milk banks in their response to novel pathogens and other emergencies.

## CONFLICTS OF INTEREST

All authors work in human milk banks. The salary of the lead author is paid by a UKRI Future Leaders Fellowship at Imperial College London, and she has no financial conflict of interest related to the milk bank in which she works.

## CONTRIBUTIONS

NS, MS, AC, PR, KIB, KM, JBvG, MBH, DK and GW drafted the manuscript from virtual discussions with the other authors. MS, KIB, KM, RCS and GW coordinated the data collection regarding the number and scale of milk bank services. RMM, VC and AW provided expert input regarding virology and screening. AV, JA, ST and SN coordinated and collated the collection of regional experiences. All authors and VCN contributors read and approved the final manuscript.

## Supporting information


**Data S1.** Supporting Information.

**Data S2.** Supporting Information
