# Supplementary material for: Maintaining human milk bank services throughout the COVID‐19 pandemic: A global response
Source: Matern Child Nutr. 2021 Jan 6;17(3):e13131. doi: 10.1111/mcn.13131 (PMC7883204; doi:10.1111/mcn.13131)
Supplement: Supplementary file 1 — Data S1. Supporting Information. [file MCN-17-e13131-s002.docx]

**Supplemental Information**

**Country-specific experiences and links to local/national guidelines collected up to mid-May 2020**

**Australia**

*Gillian Opie, Mercy Health Breastmilk Bank, Melbourne*

We have taken a specific approach given the absence of definitive evidence regarding SARS-CoV-2 and pasteurisation. At our HMB, in addition to meeting all the usual criteria, we are being extraordinarily cautious and ‘quarantining’ the breast milk of approved donors 3 weeks after expression. At 3 weeks, we check that the donor and her immediate family are well before dispensing pasteurised milk. A COVID-19-positive donor is resigned immediately and her milk discarded. We acknowledge this is an extremely cautious approach. We are finding an increase in donor applications.

*Vanessa Clifford, Laura Klein, Christine Sulfaro: Australian Red Cross Lifeblood, Level 3, 417 St Kilda Road, Melbourne, VIC, Australia*

Lifeblood Milk began in September 2018 as a service of Australian Red Cross Lifeblood, the national blood service for Australia. Lifeblood Milk is not affiliated with a specific hospital but collects and processes breast milk in New South Wales and South Australia to provide pasteurised DHM to 11 neonatal intensive care units across New South Wales, South Australia, and Queensland.

In response to COVID-19, Lifeblood Milk has made changes to the process of screening donors and collecting donations but continues to collect from both community and hospital donors. Additional screening questions have been added to identify donors with potential COVID-19 exposure or symptoms prior to any donor visits. Donors with confirmed COVID-19 are deferred for 4 weeks from the date of recovery. Donors with epidemiological risk factors for COVID-19 (close contact, travel history, etc.) are deferred for 21 days. To decrease face to face time spent with donors, all donors (new and returning), have the Donor Questionnaire and additional coronavirus questions conducted over the phone. Donor coordinators also spend more time on the phone with donors providing education that would usually be provided during the face to face interview. An appointment is then made for a Donor Coordinator to visit the donor at their home or the hospital (if the baby is still in the NICU or special care nursery). Donor Coordinators are considered an essential service, but hospitals restrictions around visitors vary – our Donor Coordinators are not permitted to enter the NICU in some hospitals.

Donor Coordinators have found direct interaction time with donors has decreased, visits with new donors previously were around one hour and now take about 20 minutes (including a blood draw for serology tests). Visits to returning donors are shorter and the Donor Coordinator can remain outside the home. During the direct interview, the donor reviews the questionnaire to ensure it is correct, provides identification, signs the paperwork (single use pen) and then packs the milk into a shipper with cold packs. Donor feedback about these changes is positive. Donors have concerns but want to continue to donate their milk and are happy with the current process.

No changes have been made to the way milk is processed, but additional Lifeblood staff have been upskilled as Milk Bank Technicians as part of the business continuity plan. Lifeblood Milk staff have always worn full sleeve gowns in the milk bank and gloves are worn whenever milk is handled. When processing, technicians also wear eye protection, a surgical mask, and a hair net. All products are hard frozen except when processing and thawed milk from a single donor is pooled to create a batch within a laminar flow cabinet before milk is Holder pasteurised in its final packaging. Lifeblood Milk has sufficient pasteurised DHM in stock to meet expected needs for at least two months.

**Brazil**

*João Aprigio, Professor, Coordinator, Ibero-American Human Milk Bank Program; National Milk Bank Service of Brazil; Fernandes Figueira Institute, Oswaldo Cruz Foundation - FIOCRUZ, Ministry of Healthl*

For our HMBs, we are working on several fronts: 24-h online technical support *via* the Internet, and communication *via* television, newspapers, radio stations, community radio and social media. Soon, in Brasília, a report will be presented on Rede Globo de Televisão with instructions to the population on the importance of donating human milk and clarifying to everyone that the process is safe and does not represent a risk for donors or those who work in home collection.

**Canada**

*Frances Jones, Coordinator, BC Women's Provincial Milk Bank, Vancouver BC Canada*

There are three milk banks in Canada. The BC Women's Provincial Milk Bank, with a provincial birth rate 44,000/yr serves over 4,000 infants per year with increasing demand every year. We solved the milk drop off issue with "contactless" drop offs to our depots (we have 28 depots that accept milk and ship to us) but are struggling with blood screening for new donors. Our Public Health Department has issued a stay-at-home order and we have been told prospective donors should not be asked to go to the hospital or community blood labs. Currently we are working on a "work around" such as having blood drawn from prospective donors at the bedside in hospital or when mothers go for follow up blood test postpartum. Unfortunately we do not have funds to have lab techs go to donors' homes.

*Janette Festival, Executive Director, Northern Star Mothers Milk Bank, Calgary, Alberta, Canada*

We are Canada’s only community-based milk bank. We are a stand-alone operation and serve 33 hospitals in 9 provinces, outpatients and pharmacies. In 2019 we dispensed 225,000 ounces. We have seen a slight decrease in donors but are able to off-set this with the help of Awareness Campaigns via social media. We have 11 ‘Milk Drops’ where mothers can drop off their donations. Two of the milk drops are located in hospitals. To reduce hospital exposure we have charitable organizations sweep the city and pick up the donations which are then dropped off at the milk drops by one person. We are fully operational with lab staff working overtime to pasteurize as much milk as possible – we also serve as a back-up for other milk banks. If a mother is suspected of having COVID or is COVID-positive, the Canadian Pediatric Society has recently released a statement giving guidance on how to safely breastfeed:

“Women who choose to breastfeed should wear a mask (if available), wash their hands, and clean their breast area with soap and water before each feeding. Mothers may also choose to pump - ensuring that they wash their hands and clean all equipment - and then feed their infant expressed breastmilk. At home, frequently touched household surfaces should also be disinfected regularly. Symptomatic individuals should not be allowed to visit with mother and baby.

If a mother is too sick to breastfeed, due to COVID-19 or other complications, she should be encouraged and supported to express milk. Pumped breastmilk can safely be supplied to the infant, as long as appropriate local infection prevention and control (IPC) guidelines are followed. If institutional IPC policies prevent NICU visitation by mothers with suspected or proven COVID-19, those mothers should be encouraged to pump at home and provide expressed breastmilk to the NICU. Mothers should pump frequently at home, with a view toward achieving successful breastfeeding once she is able to visit her baby.

**China**

*Xihong Liu, Medical Director, Guangzhou HMB, Guangzhou*

The pasteurisation process for donated milk was not changed in some human milk banks in recent months. But, most human milk banks have stopped donation since 2 months ago. Anyway, the need and use decreased much. It is not difficult for us to find donors because the donors who come to our human milk bank have large amounts of milk. We did not check the COVID-19 virus in donor milk specifically. Donor screening became very strict, like epidemiological history inquiry, commitment signature, temperature measurement, and previous examination items.

**Denmark**

*Anne Bille Olin, Lead Clinical Dietician, Children’s Department, Women’s Milk Center, Hvidovre Hospital, Kvindemælkcentralen afs. 529, Kettegård Allé 30, 2650 Hvidovre.*

There has been no change to services in Denmark. We have a lot of milk in stock and we have lot of donors who want to help. In Denmark, the breastfeeding rate is very high. Instead of a face-to-face consultation for new donors, we now talk over the phone and the mothers go to a nearby lab for screening tests. Before that, they came to the hospital for the screening and blood tests. We have drivers that pick up the milk from donors. The donors put it outside the door and the drivers pick it up. Every donor has her own transport bag and each donor’s milk is packed in separate cool bags in the van. All DHM is kept for at least 4 weeks before it is used for NICUs at the hospital or to other hospitals in Denmark as well as Iceland, Greenland and the Faroe Islands. In Denmark mother’s own milk is prioritized and after that DHM is used until the baby reaches 1500 g.

**France**

*Prof Jean-Charles Picaud, Virginie Rigourd, Cécile Boscher, Rachel Buffin, Fouzia Voirin, Solène LEFUR Board of the French Human Milk Banks Association (Association des Lactariums de France, ADLF)*

COVID-19: Position of the French Human Milk Banks Association (ADLF, abridged)

In the current epidemic context, questions arise concerning breast milk donors and the treatment of breast milk in human milk banks.

• Regarding human milk and breastfeeding: Breast milk is absolutely essential for premature infants, as it significantly reduces the risk of serious complications related to prematurity, in the short and long term. There are major health benefits for these high-risk infants. Breastfeeding is allowed in mothers with COVID-19, unless they have a severe infection that seriously affects their health. Breastfeeding must be carried out under strict hygienic conditions. Based on current data, SARS-CoV-2 is not present in breast milk. SARS coronaviruses are sensitive to heat. It is very likely that SARS-CoV-2 would be inactivated by pasteurization (62.5°C for 30 min) used in human milk banks.

• Regarding breast milk donation and human milk banks: This is organized and supported by the French human milk banks which all respect common rules concerning the selection of donors based on a health questionnaire and on blood sampling (hepatitis, HIV, HTLV), as well as the handling and treatment of milk by Holder pasteurization, in accordance with the official Guide to good practice published in the official journal of the French Republic (Guide to Good practice 2008). The hygiene rules applied in French milk banks protect against transmission of the virus. Donors are well informed about the importance of adhering to strict hygiene rules when collecting and transporting milk. Specific questions were added to the usual health questionnaire, in order to define whether the donor is "at risk", that is to say if she has symptoms suggestive of COVID-19 (fever, headache, severe asthenia, cough, diarrhoea, vomiting, anosmia, etc.) or a confirmed diagnosis of COVID-19. In this case, the ADLF recommends temporarily suspending the donation (or not recruiting) for 14 days, or up to 7 days after the symptoms have ended.

• Regarding the treatment of milk from a mother to her own infant: Since breastfeeding is considered possible in COVID-19 or suspect mothers, human milk banks can continue to pasteurize this own mother's milk when needed.

*Rachel Buffin, Neonatologist, Human Milk Bank, Neonatal unit, Hospices Civils de Lyon, Hôpital de la Croix Rousse, F-69004 Lyon*

Breastfeeding (even in a neonatal unit) is encouraged with symptomatic mothers wearing a facemask and involves bottle disinfection if she is pumping. We quarantine for 14 days and check on the mother by telephone. Before each collection, we call the donor and report if she has presented symptoms. We keep 2 m from the donor and disinfect the ice box holding the DHM. Currently, donors can continue to visit the laboratory.

**Germany**

*Daniel Klotz, Head of Neonatology, Freiburg University Hospital, Freiburg*

Official recommendations concerning the use of DHM are not available. Currently DHM programs are not being suspended but routine handling practices concerning the acceptance of milk donations are being modified to meet hygienic recommendations issued in the context of the COVID-19 epidemic. However, Germany is one of the few countries where unpasteurized DHM is dispensed by some units and most of these units have currently stopped this practice. There are some concerns about the safety of pasteurization for Sars-CoV-2 leading to discarding of milk of mothers suspected of or suffering from COVID-19. Neonates and COVID-19 positive mothers were not separated systemicatally and breastfeeding is still encouraged but in the early days of the pandemic some hospitals barred partners of pregnant mothers from attending the birth of their children due to “visitor” regulations. The widespread closure of maternity wards for visitors, which includes the newborn fathers, increased post-partum stress and led to an increase of ambulatory deliveries. The concurrent reduced access to ambulatory care may lead to impaired post-partum care to newborns and their mothers. Most information is currently anecdotal or from personal communication and needs to be systematically collected to assess the extent of potential unintentional collateral damages of the COVID-19 response in this regard.

**India**

*Jai Singh, Neonatologist, President, Monitoring and Mentoring Committee, Human Milk Banks, Rajasthan*

As before, all mothers are being counselled to breastfeed but with adequate precautions, including handwashing and masks. Mothers of sick babies who can’t breastfeed are supported to provide EBM. Generally in the first 2-3 days, milk production is less in these mothers, so we provide DHM to bridge the gap and then once the mothers start lactating sufficiently, babies go back to being fed mothers’ milk. Right now, DHM supplies are deficient. We outsource the milk culture, and so as a result of the lockdown and lack of transportation getting milk to the lab is an issue. We have had to completely stop collecting milk. But with PATH’s support we are as of yesterday collecting and storing milk again (can be stored up to three months) and we will begin the culture process once things get back to normal. We are rationing the DHM for very small and sick babies. The number of deliveries have also reduced by 35- 40%, so demand has lessened too. Mothers are scared and want to avoid hospital visits as much as possible. Health care workers are facing challenges – there are knowledge gaps and lack of awareness on guidance on infant feeding. PPE deficiency is also a problem though not as widely.

Mothers are open to donating but we are screening them more rigorously and asking them additional questions about COVID-19. We haven’t had a single positive case among pregnant women in Rajasthan, but if we have we will follow the WHO protocol and encourage these mothers to breastfeed taking all precautions. If for any reason they need to be separated we will encourage expression. However, mothers are scared and confused. They require adequate information on infant feeding practices during these times.

Our key message is that breastfeeding should not be stopped because of Covid challenges and every effort should be made to ensure the needy babies receive DHM. so proper rationing should be practiced.

*Professor Sushma Nangia, Neonatologist, Vatsalya Maatri Amrit Kosh, Lady Hardinge Medical College, New Delhi*

The National Human Milk bank is providing screened DHM to babies admitted within the hospital NICUs and the Neonatal Covid facility. In addition, we are supporting one hospital’s NICU by pasteurizing their collected milk in a hub and spoke model. Two more Institutions are being added for similar support. In addition, two NICUs of one and only exclusive COVID hospital in Delhi will be supported by us for providing PDHM to the neonates admitted there.

*Suchandra Mukherjee, Neonatologist, Kolkata*

Screening of donors has become rigorous with additional questions on COVID being asked, including questions on symptoms, travel history, and contact history. Initially we were not allowing mothers in the community to donate, but after a 14-day incubation period has passed we are now recruiting them. Initial shortages of milk bank staff, panic and fear and lack of accurate information on Covid among staff brought challenges. Mothers also need to be counselled regularly and informed of precautions especially after discharge. We are providing masks to health workers, but mothers have to buy them from outside. We have to make sure they use masks. Symptomatic mothers can feed MOM to their babies but not allowed to donate. The hospital is more crowded since patients from other Covid-specific hospitals are coming here, with 10-12 extra deliveries per day.

Regarding donation, staff could not come into the milk bank for a week because of transportation issues. Two nursing staff are now staying at the hospital to help with donor recruitment. Outpatient services are scanty, and the number of mothers attending has dropped, so donations have reduced. We are now rationing DHM. We give it to level III babies and level II babies we give cow milk or formula milk.

For positive cases, if mother is asymptomatic they can be put together with the baby isolated from other mothers and mothers can breastfeed. If mother is sick, baby to be isolated and fed expressed milk or donor milk by a relative who has not come in contact with mother during the incubation period. Kolkata has fewer cases, so it is not as much of a concern as is the case with states with higher case loads. There is a significant need for counselling health care workers and mothers to address their knowledge gaps and fears. We are preparing a booklet to address these concerns. Protocols should be clear and well communicated to health care workers.

*Kajal Jain, Neonatologist, Human Milk Bank Lead, AIIMS, Delhi*

The footfall has reduced greatly. We have discharged all babies except the very sick ones. Initially three babies were being given DHM. Staff of CLMC are working from home to avoid exposure. Mothers are not donating as there is no staff to help. The two counsellors used their private vehicles to come for a day as the stored donor milk (45 litres) had to be sent for pasteurization to Lady Hardinge hospital before it expired. This is now enough to serve our need. As the lactation counsellors are not now in the hospital, we have kept 10 bottles of DHM in upper chamber of refrigerator so that NICU nurses can thaw and give when needed. The lactation counsellor who lives nearby can come and place more bottles when the current lot finishes.

One mother has tested positive and so is the father. The baby is term and has been roomed in with mother and being breastfed with mother taking all precautions. Mother has been isolated from other mothers. The baby has not been tested. Lactation counsellors are calling mothers regularly and counselling and guiding them on breastfeeding and any associated COVID fears and precautions. If they have any medical concern, they are asked to call the NICU nurse and the doctor and check with them before coming to the hospital. Most cases are being dealt with telephonically.

Only pediatric emergency care is functioning. Before any patient is referred to a ward, they are screened for COVID, including being asked questions on travel and contact history. Vaccination of babies is the biggest challenge, since hospital visits other than medical emergencies has been stopped. Mothers are told to get their babies vaccinated from local clinic or Mohalla clinics. NICU nurses want to stay at the hospital as they do not want to expose their family members to any risk. Discussions are ongoing to arrange for their stay at the hospital hostel.

Mothers should be breastfeeding their babies as much as possible. They should minimize their visits to hospitals at such times and get help telephonically from doctors. A small 2-3 mins video on breastfeeding, positioning and latching is helpful being shared with mothers over WhatsApp.

*Suksham Jain, Neonatologist, Government Medical College Hospital, Chandigarh*

All breastfeeding, breastmilk expression, kangaroo mother care and skin-to-skin contact are continuing as usual. We have enough breast pumps to ensure the hygienic expression of milk. Only pooling and pasteurization has stopped. The responsible staff for this this lives in another state and her commute has been impacted by the lockdown. We have hired another person now, but she is yet to be trained properly. We are developing transport contingencies. As the hospital’s microbiology lab is overburdened with COVID-19 testing they do not have sufficient time to do the milk culture. We don’t want to burden them during this trying time. Our delivery load and number of babies admitted have decreased. We are only keeping high risk babies in the NICU and step-down area. Demand for donor milk is lower and we have sufficient supply to address the need. We are taking steps to restart donor milk processing.

The lactation counsellors were coming to work on alternate days because of lockdown but now they are coming every day. We have stepped up counselling so that all babies get mothers’ milk. We are also practicing relaxation techniques with mothers - listening to music and religious book reading sessions. We were planning on magazine subscriptions too but that has been put on hold because of lockdown. We want mothers to be relaxed and happy so that they can produce more milk. Mothers are upset about COVID but none have asked to be discharged, possibly because they feel safer at the hospital. I have asked the counsellors to talk to mothers about their concerns. We need clear guidance from the government of India on this as we have to strike a balance between risks of contracting COVID versus ensuring proper nutrition for babies. We also need extra staff to ensure proper hygiene while these mothers feed their babies. Counselling mothers to breastfeed and keeping them happy and relaxed should be the top priority to improve milk supply for babies.

**Iran**

*Mohammadbagher Hosseini, Neonatologist, Human Milk Bank of Tabriz University Hospital*

Iran has been affected deeply by COVID-19. We have nine active HMBs. Fortunately, the Ministry of Health has released several guidelines for the diagnosis and treatment of COVID-19. One of the guidelines focused on breastfeeding and milk donation for confirmed and suspected cases of COVID-19. Before the pandemic, in our HBM Bank in Tabriz, we pasteurised around 4.5–6.5 L of DHM every day. Currently, we are pasteurising ~2.5 L of DHM because of a reduction in the number of donors. A similar scenario has arisen in Shahid Akbarabdei Hospital in Tehran.

**Ireland**

*Tanya Cassidy, School of Nursing, Psychotherapy, and Community Health, Dublin City University*

A poorly timed *Irish Times* article has said that top-ups with formula, especially during the COVID-19 crisis, is perfectly fine, and that the evidence-based research regarding health benefit/harms is limited. These are often the same arguments we hear from the formula industry undermining breastfeeding.

**Kenya**

*Mary Waiyego, Neonatologist, Pumwani Maternity Hospital Lactation Support Centre and Human Milk Bank, Nairobi*

Pumwani Maternity Hospital Human Milk Bank has been in operation for just over a year. Human milk donors are recruited from mothers who are hospital inpatients. The recipients are vulnerable and eligible babies admitted in the hospital’s new-born unit. The process of screening mothers involves administering a risk assessment questionnaire, screening blood for potentially transmissible infection. We then use Holder pasteurisation for the donated screened milk.

The first case of COVID- 19 was confirmed in Kenya on the 13^th^ March, 2020. Since then there are a total of 225 reported cases as of 15^th^ March, 2020. Community transmission has been recorded.

There is heightened screening of all mothers at the entrance to the hospital and during the process of admission. The screening also includes all the visitors to the hospital and the people accompanying mothers to the hospital. In addition to mothers being screened prior to admission, donor mothers are also getting additional COVID-19 screening questions at the point of recruitment.

Other addition infection prevention measures include: emphasising hand hygiene, the use of surgical masks in both donors and human milk attendants, wiping the outside of containers with 0.5 sodium hypochlorite. Human milk bank services continue whilst applying these measures.

**Myanmar**

*Nant San San Aye, Head of Neonatology, Central Women’s Hospital, Yangon*

We opened our Standardized Human Milk Bank on the 11^th^ January 2020 following the relevant Standard Operating Procedures and Myanmar National Guideline. Before 2020, we did not collect data regarding the number of donors recruited from the Lactation Management Clinic (LMC) or the postnatal wards. To avoid overcrowding during the COVID-19 pandemic, we closed the LMC as well as the Baby Clinic from 1^st^ April 2020, which usually opened weekly. We still collect the milk from the postnatal wards. Our hospital guideline currently separates women with fever onto a separate Fever Ward. There have been no suspected or confirmed cases of COVID-19 cases to date. As there is a long holiday from 10^th^ April to 19^th^ April (Water Festival and New Year Festival), staff shortages are usual.

**Norway**

*Anne Hagen Grøvslien, Milk Bank Manager, Oslo University Hospital*

There has been no change to DHM services in Norway so far and most of the DHM continues to be fed raw (i.e., without pasteurisation). We have sufficient DHM for NICUs and a sufficient number of donors. More potential donors are contacting us, wanting to help. All DHM is ‘quarantined’ until we call the donor after 14 days to ensure symptoms have not developed. It seems that society has changed here. People are offering help, food, and all sorts of service for people in need. Mothers with extra milk are offering it. Guidelines for Norway were published on 20^th^ March 2020:

<https://oslo-universitetssykehus.no/fag-og-forskning/nasjonale-og-regionale-tjenester/nasjonal-kompetansetjeneste-for-amming-nka>)

**Philippines**

*Estrella J. Olonan-Jusi, Human Milk Bank Director, President, Human Milk Bank Association of the Philippines, Dr. Jose Fabella Memorial Hospital*

The COVID-19 pandemic has a significant impact on the supply of donor milk in the country because mothers can’t go to the milk bank to donate or the HMB staff can’t go to the community especially during this time that enhanced community quarantine is implemented and public transportation is suspended. Therefore, we can only recruit donors among mothers who delivered in the hospital. In our hospital, the patients classified as patients under investigation are admitted to a separate building. For those mothers in our rooming-in ward, still we are asking potential donors the questions to screen for exposure to PUI or COVID-19 confirmed cases, history of fever, cough and cold. The hospital provides mothers with face masks and the HMB staff wear face mask, goggles and gloves when interviewing and assisting the mothers with breast milk collection in addition to the recommendations for hand hygiene and hand washing. We are limiting DHM for critically ill babies because our stocks may become depleted. Our country is in the second week of enhanced community quarantine. Public transportation is suspended temporarily so people must stay at home. Breastfeeding is recommended for mothers who are under investigation or confirmed to have COVID-19 with an emphasis on hygiene measures and wearing of a facemask when breastfeeding.

**Poland**

*Aleksandra Wesolowska, Human Milk Bank Foundation, Holy Hospital, Medical University of Warsaw, Warsaw, Poland*

In Poland, we follow the practice in China: delivery of babies from mothers infected (or suspected of being infected) with SARS-CoV-2 must be by Caesarean section. Mothers infected (or suspected of being infected) with SARS-CoV-2 are not allowed to breastfeed or feed the baby expressed mother’s milk. The National Neonatology Society has stated that newborns could be fed by their own mother’s milk if pasteurised, which is unrealistic in the current circumstances. Mothers are encouraged to maintain lactation but expressed milk should be discarded until mothers have two consecutive negative SARS-CoV-2 test results. In Poland, this recommendation has raised concerns among mothers and NGOs. There is DHM in stock; however, due to logistical and hygienic reasons, its use has been limited during the pandemic. Medical staff in hospitals wish to avoid unnecessary risks, especially with the current shortages of PPE. Supporting lactation in COVID-19 infected mothers during the pandemic is considered an unnecessary risk.

**Serbia**

*Radmila Mileusnic-Milenovic, Neonatologist, First Serbian Human Milk Bank, Institute of Neonatology, Belgrade*

There are no recommendations for MOM in Serbia. At the Institute of Neonatology where I work, there are about 700–800 very premature neonates. All visits have been suspended and MOM is not available. Only a few mothers have taken shelter in our hospital. With regard to the HMB, 10 days before the pandemic, our pasteuriser broke down, so all activities have ceased. The epidemic is in its third week. All mothers are in telephone contact or have been confirmed to have COVID-19 at this time.

**Spain**

*Nadia Garcia-Lara, Neonatologist, 12 Octubre Hospital Regional Milk Bank, Madrid*

Although initially mother-child dyads for confirmed/suspected COVID-19 were separated in most hospitals, the number of maternity wards that are promoting cohabitation, skin-to-skin contact and breastfeeding are increasing quickly. We have stablished specific temporary exclusion criteria for confirmed/suspected COVID-19 similar to blood donors. At the moment, donor milk use is prioritized for more vulnerable infants. Home collection systems are fully working (special safety standards developed) A new initiative is working in Granada for home collection (volunteer taxi drivers). Some restrictions in recruitment to avoid women coming to hospitals, although probably recruitment will be restarted with safety standards at the personal interview at milk bank.

We stopped recruiting donors at the beginning of March to avoid donors coming to hospital. We maintained recruiting donors at hospital environment (women that have surplus milk and have their own newborn admitted in the Neonatology Unit). In a short time, it is planned to restart recruiting donors (previous telephone interview and then personal interview at milk bank or at home by a nurse following safety standards). Thanks to a home milk collection programme funded by Ronald McDonald Charity Houses, we have plenty of DHM. It was an agreement signed in March 2019 from our regional HMB and Ronald McDonald House Charities in Spain.

**South Africa**

*Jenny Wright, Director of Milk Matters, Human Milk Bank, Cape Town*

Our milk bank remains fully operational with additional screening of donor mothers for COVID-19 infection or exposure, plus extra safety measures in place when collecting donor milk from donors or depots (milk donation drop off points in the community), when donor milk is collected by hospitals and in the staffing of our milk bank. The workload for staff has increased due to the additional measures instituted as well as no longer allowing volunteers in high risk groups (e.g., senior citizens) to be involved.

Although a very strict lockdown is in place in South Africa, human milk banking and breast milk donations are deemed to be essential services, so mothers are allowed to travel to drop off breast milk donations or have the required blood tests done. As it is relatively early in the pandemic in South Africa, it remains to be seen how donations of breast milk, donors going for the tests and prescribing of pasteurised donor breast milk are going to be affected. Appeals for mothers to register to donate breast milk are being made in conjunction with assurances about the safety of donor breast milk in efforts to maintain, and ideally increase, our supply. Concerns remain however that supplies will be under pressure in the weeks to come. Any interruption in supply will have a severe impact as the donor milk recipients are low birth weight and very low birth weight premature babies without access to sufficient breast milk from their own mothers.

**Sweden**

*Josefin Lundström, Neonatologist, Head of Milknet, the Swedish milk banking network.*

In Sweden our existing guidelines included strict hygienic instructions and guidelines regarding viral infections. We therefore felt no need to stop human milk donations. We have complemented our guidelines with stricter information regarding viral infections with only slight symptoms and for a mandatory 48 hours to pass after experiencing any viral symptoms. Our personnel collecting donor milk at the homes of donors phone ahead and the donors leave the milk outside minimizing contact between personnel and donors.

During the COVID-19 epidemic, in Sweden, donating mothers are allowed to travel to the milk bank but generally do not. They are reluctant to come to the hospital and also reluctant to have blood tests taken even if they are taken outside of the hospital. Therefore, donors have decreased by at least 50% and most of the donated breastmilk, at least in Stockholm, is donated by mothers having infants in the neonatal ward. Mothers that are SARS-CoV-2 positive are not separated from the infants and are allowed to breastfeed as usual, but while wearing a mouth mask.

**Taiwan**

*Florence (Leefung) Funglee, Neonatologist, Taiwan Children’s Hospital Milk Bank (TCHMB)*

We are proceeding as usual, though a small reduction in DHM has been noted. We follow our own standard operating procedures strictly. We emphasise appropriate handling of DHM is very important to our donors and recipients. We do not have specific guidelines for COVID-19, but the national movement against COVID-19 is strong.

**Thailand**

*Sopapan Ngerncham, Neonatologist, Associate Professor, Division of Neonatology, Department of Pediatrics, Faculty of Medicine Siriraj Hospital, Mahidol University, Bangkok*

There are four HMBs operating at the moment. Siriraj and Ramathibodi HMB are in Bangkok. Songklanagarind HMB is in Songkla province in the south of Thailand and they started their service in October 2019. The last one is Chiangmai HMB in Chiangmai province in the north of Thailand. Chiangmai HMB just started a running test, so they don't have any recipient of pasteurized donor human milk yet. At Ramathibodi and Songklanagarind HMB, they have also stopped recruiting new milk donors at the moment.

Since early this year, we started to find confirmed COVID-19 cases in Thailand. From early-March, the number of cases increase dramatically due to a few specific events in the country. Since then, the government asked people to stay at home and the policy is getting more serious. We are now in the curfews between 2200-0400. In Bangkok, we do not encourage the mothers to come to the hospital until the local situation is better. We still have some pasteurized DHM in stock, which hopefully will last for another month.

**UK**

*Gillian Weaver, Cofounder, Human Milk Foundation, Rothamsted Institute*

Having recognised the potential for pandemic in January, we consulted with our expert advisory team and put out guidance regarding donor screening for milk banks on the 23^rd^ January, to which we have added as new data became available. At Dr Shenker’s instigation, UK milk banks have since been working together using weekly calls to discuss new issues and develop mitigation strategies, underpinned by daily communications through WhatsApp. Data collection tools have been disseminated to facilitate preparedness. A call for donors and volunteer phlebotomists was put out through social media and the BBC, with an excellent response, but sufficient numbers of donors have come through without needing to adopt unusual measures at the moment. All milk banks are still operating in accordance with the NICE Clinical Guideline, and have agreed that deviations from this guidance would only be considered if a) DHM supplies ran critically low and b) consensus was reached between all milk bank leads.

*Jackie Hughes, Chair of UKAMB (UK Association of Milk Banking); Director of the Northwest Human Milk Bank*

We have just built-up a 2-week stock of DHM. We will ‘quarantine’ DHM in a separate room for 14 days from now. We are telephoning the donors of human milk that has been pasteurised to check that they are free of symptoms. We follow the recommendations from the UNICEF UK Baby Friendly Initiative: [www.unicef.org.uk/babyfriendly/infant-feeding-on-neonatal-units-during-the-covid-19-outbreak/](https://www.unicef.org.uk/babyfriendly/infant-feeding-on-neonatal-units-during-the-covid-19-outbreak/)

*Debbie Barnett, Milk Bank Lead, Scottish National Milk Bank Service, Glasgow, Scotland.*

We screen the donor and her household to ensure they have been symptom-free for 14 days.

**USA**

*Pauline Sakamoto, Director, San Jose Mother’s Milk Bank, CA; HMBANA*

We are continuing to operate at full capacity. Like blood banking in the USA, we are anticipating a slowdown in donation of human milk soon. However, hospitals and families continue to demand DHM. We are staggering staff rotas to ensure social distancing of 2 m in the facility. We have increased screening to include COVID-19 by asking questions about personal contact.

We have local donors dropping off human milk at our facility, but most donation is by overnight shipment. We have suffered cancellations of milk drives in various communities in California. However, our hospital-based milk collection sites/depots continue to collect milk or provide shipping containers to mothers. The biggest fear is going outside the home and becoming infected during phlebotomy.

*Naomi Bar-Yam, PhD, Mother's Milk Bank Northeast, 377 Elliot St, Newton Upper Falls, MA 02464*

We have addressed the thorny issue of triaging. Huge potential upcoming challenge for HMBs: turning down requests for DHM next week that would have been agreed last week.
