# Supplementary material for: Maintaining human milk bank services throughout the COVID‐19 pandemic: A global response
Source: Matern Child Nutr. 2021 Jan 6;17(3):e13131. doi: 10.1111/mcn.13131 (PMC7883204; doi:10.1111/mcn.13131)
Supplement: Supplementary file 2 — Data S2. Supporting Information [file MCN-17-e13131-s001.docx]

**Virtual Collaborative Network of Milk Banks and Associations contributors:**

| **Country** | **Name** | **Credentials** | **Role** | **Address** |
| --- | --- | --- | --- | --- |
| Australia | Christine Sulfaro |  | Milk Bank Manager | Australian Red Cross Lifeblood, Level 3, 417 St Kilda Road, Melbourne, Victoria, Australia |
| Australia | Gillian Opie | MD | Neonatal Paediatrician, Head of Unit | Mercy Health Breastmilk Bank, Melbourne Australia |
| Australia | Laura Klein |  | Research Fellow | Australian Red Cross Lifeblood, Level 3, 417 St Kilda Road, Melbourne, Victoria, Australia |
| Austria | Andreas Malzacher | MD | Director |  |
| Canada | Frances Jones | RN, MSN, IBCLC | Executive Director, Past President, HMBANA | British Columbia Women's Mothers' Milk Bank, Vancouver, Canada |
| Canada | Janette Festival | RN, INCLC | Executive Director | NorthernStar Mothers Milk Bank, Calgary, Alberta, Canada |
| China | Xihong Liu | MD | Director of Clinical Nutrition | Guangzhou Women and Children Medical Center |
| Croatia | Branka Golubić-Ćepulić | MD | Head of Department of Transfusion Medicine and Transplantation Biology | Department of Transfusion Medicine and Transplant Biology, Clinical Hospital Centre, Zagreb |
| Denmark | Anne Bille Olin |  | Lead Clinical Dietician | Children’s Department, Women’s Milk Center, Hvidovre Hospital, Kvindemælkcentralen afs. 529, Kettegård Allé 30, 2650 Hvidovre |
| Estonia | Annika Tiit | MD | Director | Human Milk Bank, East Tallinn Central Hospital |
| France | Claude Billeaud | MD, MSc Dr of Science and Nutrition | President AEEP, Asst Clin Director of Paediatrics, Scientific Manager Bordeaux Marmande Human Milk Bank | Association Européenne pour l'Enseignement en Pédiatrie, University of Bordeaux |
| France | Rachel Buffin | MD, Neonatologist | Neonatologist in Charge of the Lactarium/Human Milk Bank | 13 Auvergne Rhone Alpes regional Human Milk Bank, Lyon , France; Médecin du Lactarium Régional Rhône Alpes, Hôpital de la Croix Rousse, 103, grande ruse de la Crois Rousse, 69317, Lyon Cedex 04 |
| India | Adhisivam Bethou | MD | Ass Prof and Head of Dept | Dept. neonatology JIPMER, Pondicherry 605006 |
| India | Himabindu Singh | MD | Clinical Director | Osmania Medical College |
| India | Jai Singh | MD | Clinical Director | President, Monitoring and Mentoring Committee, Human Milk Banks, Rajasthan |
| India | Jayendra Kasar | MD | Clinical Director | Centre for Health Research and Innovation (CHRI) |
| India | Kajal Jain | PhD | Human Milk Bank Lead | AIIMS, Delhi |
| India | Ketan Bharadva | MD | President-elect | Human Milk Banking Association (India); Human Milk Donation Camps of Surat Pediatric Association, India. |
| India | Poonam Singh | MD | Clinical Director | Neonatologist, Surat Milk Bank, Gujarat |
| India | Pratibha Kale | MD | Clinical Director | Human Milk Bank, Amrivati |
| India | Roopa Bellard | MD | Clinical Director | NJ Medical College, Karnataka, India |
| India | Sila Deb | MD | Deputy Commissioner, Child Health | Ministry of Health and Family Welfare |
| India | Suchandra Mukherjee | MD | Clinical Director | Human Milk Bank Lead, Kolkota |
| India | Suksham Jain | MD | Neonatologist | Government Medical College Hospital, Chandigarh |
| India | Sushma Nangia | MD, DM | Convener, National Human Milk Bank; Director, Professor & Head of Department | Department of Neonatology, Lady Hardinge Medical College & Kalawati Saran Children's Hospital, New Delhi 110001 |
| India | Selvaraj Jayaraman | MD | Professor, State Advisor | Saveetha Hospital, Tamil Nadu |
| Iran | Mohammad Heidarzadeh | MD | Director of Neonatal Office | Ministry of Health; Tabriz University of Medical Sciences |
| Iran | Maryam Saboute | MD | Clinical Director | Human Milk Bank of Shahid Akbarabadi Hospital of Tehran |
| Ireland | Tanya Cassidy | PhD, MA |  | School of Nursing, Psychotherapy, and Community Health, Dublin City University, Ireland |
| Italy | Enrico Bertino | MD | President European Milk Bank Association, Prof of Neonatology | Università degli Studi di Torino \| UNITO · Dipartimento di Scienze della Sanità Pubblica e Pediatriche |
| Italy | Guido Moro | MD | Professor of Neonatology (ret), President AIBLUD | Italian Association of Donated Human Milk Banks (AIBLUD); Biomedia, Via Libero Temolo No 4, 20126, Milan, Italy |
| Kenya | Angela Kithua | MSc (Nut) | Nutrition Program Associate | PATH, supporting the Pumwani Maternity Hospital, Kenya |
| Kenya | Faith Njeru | RN | Paediatric Nurse | Pumwani Maternity Hospital Lactation Support Center and Human Milk Bank, Nairobi, Kenya |
| Kenya | Mary Waiyego | MD |  | Pumwani Maternity Hospital Lactation Support Center and Human Milk Bank, Nairobi, Kenya |
| Myanmar | Nant San San Aye | MD | Professor of Neonatology | Central Women’s Hospital, Yangon |
| Myanmar | San San Myint | MD | Former Prof Neonatology, Founder Milk Bank, CWH | Central Women’s Hospital, Yangon |
| Myanmar | Zaw Win Moe | MD |  | Yankin Children’s Hospital, Yangon |
| New Zealand | Anthea Franks | RN |  | Human Milk Bank, Neonatal Unit, Christchurch Women’s Hospital, New Zealand |
| Norway | Anne Bærug | PhD | Norwegian National Advisory Unit on Breastfeeding | Norwegian National Advisory Unit on Breastfeeding, Oslo, Norway |
| Philippines | Estrella J. Olonan-Jusi | MD,MPM | Human Milk Bank Director, President, Human Milk Bank Association of the Philippines | Dr. Jose Fabella Memorial Hospital, Philippines |
| Scotland | Debbie Barnett | RN | Manager | Scottish National Milk Bank Service, Glasgow |
| Slovenia | Andreja Domjan | MD, PhD | Consultant Paediatrician | Ljubljana Maternity Hospital |
| South Africa | Jenny Wright | RN | CEO | Milk Matters, Human Milk Bank, Cape Town, South Africa; Board of HMBASA (Human Milk Banking Association of South Africa) |
| Spain | Antoni Gaya | MD, PhD | Director, Tissue Bank | Fundació Banc de Sang i Teixits de les Illes Balears, Institut d’Investigacions Sanitaries Illes Balears (IDISBA), Palma, Spain |
| Spain | Nadia Garcia-Lara | MD, Neonatologist | Neonatologist | 12 Octubre Hospital Regional Milk Bank, Madrid, Spain |
| Sweden | Josefin Lundstrom | MD | Neonatal Consultant | Sachsska Children's and Youth Hospital |
| Taiwan | Florence Leefang Fanglee | MD | Medical Director, Human Milk Bank, Head of Neonatal Unit | Taipei City Hospital Milkbank |
| Taiwan | Yungchieh Lin (Apple) | MD | Medical Director Human Milk Bank, Neonatologist | Southern Milk Bank |
| Thailand | Sopapan Ngerncham | MD, MSc | Secretory of the Committee, Siriraj Human Milk Bank | Division of Neonatology, Department of Pediatrics, Faculty of Medicine Siriraj Hospital, Mahidol University, Bangkok, Thailand |
| Turkey | Sertac Arslanoglu | MD, PhD | Professor Neonatology, Vice-President European Milk Bank Association | Director, Dr Behcet Uz Children’s Hospital, Izmir, Turkey |
| UK | Jackie Hughes | RN | Chair, UK Association for Milk Banking | Northwest Human Milk Bank, Chester |
| USA | Amy Vickers | MSN, RN, IBCLC | Current President HMBANA and Executive Director Mother's Milk Bank of N Texas | Executive Director, Mothers’ Milk Bank of North Texas; President- Board of Directors- HMBANA |
| USA | Erin H Spence | MD | Neonatologist, Co-Medical Director | Mother's Milk Bank of North Texas, 7617 Benbrook Parkway, Fort Worth, Texas 76126 USA |
| USA | Laraine Lockhart Borman | IBCLC | Director of Outreach, Milk Bank | Director, Mothers' Milk Bank, Rocky Mountain Hospital for Children, 1719 E, 19th Ave, Denver CO 80218 USA |
| USA | Lindsay Groff | MBA | Executive Director | Human Milk Banking Association of North America (HMBANA), 455 Camp Bowie Blvd. Suite 114-88, Fort Worth, TX76107 |
| USA | Pauline Sakamoto | MS, RN, PHN | Operations and Regulatory Consultant, Past President | San Jose Mothers’ Milk Bank, HMBANA |
| USA | Sybil Sanchez | MIA, CLC | Associate Director | Human Milk Banking Association of North America (HMBANA), 455 Camp Bowie Blvd. Suite 114-88, Fort Worth, TX76107 |
| USA | Naomi Bar Yam | PhD | Director | Mother's Milk Bank Northeast, 377 Elliot St, Newton Upper Falls, MA 02464 |
| Vietnam | Tran Thi Hoang | MD, PhD | Vice-Director | Human Milk Bank, Da Nang Hospital for Women and Children. Ha Noi |
| Vietnam | Roger Mathieson | MSc Clinical Nutrition | Regional Director | Alive and Thrive Southeast Asia, Hanoi, Vietnam |
